# The Experienced Benefits of the 17-Item Benefit Finding Scale in Chinese Colorectal Cancer Survivor and Spousal Caregiver Couples

**DOI:** 10.3390/healthcare9050512

**Published:** 2021-04-28

**Authors:** Meizhen Chen, Jiali Gong, Jieyu Li, Xingjuan Luo, Qiuping Li

**Affiliations:** Wuxi School of Medicine, Jiangnan University, Wuxi 214122, China; 6202807003@stu.jiangnan.edu.cn (M.C.); 6202807012@stu.jiangnan.edu.cn (J.G.); 6182806003@stu.jiangnan.edu.cn (J.L.); 6182806004@stu.jiangnan.edu.cn (X.L.)

**Keywords:** colorectal cancer, spousal caregivers, benefit finding, a 17-item benefit finding scale, psychometric property, Chinese

## Abstract

The specific aims of the present study were twofold: (i) to examine the psychometric properties of a Chinese version of the 17-item Benefit Finding Scale (BFS-C), and (ii) to explore the experienced benefits in colorectal cancer (CRC) survivors and their spousal caregivers (SCs). A total of 286 CRC survivors and SCs participated in the investigation, which assessed participant variables of demographic characteristics and benefit finding (BF). Statistical methods applied were confirmatory factor analysis (CFA), Cronbach’s α, Pearson’s correlation, Kappa coefficient, paired *t*-tests, and one-way ANOVAs. CFA analysis supported a three-factor model for structure validity. All Cronbach’s α for BFS-C was greater than 0.870 in both CRC survivors and SCs. The test–retest correlations at the scale level ranged from good to excellent for CRC survivors (r = 0.752–0.922), and from moderate to good for SCs (r = 0.469–0.654). There were moderate to high correlations between CRC survivors and SCs in all of the paired BFs (all Ps < 0.001, r = 0.332–0.575). This report provided the satisfactory psychometric properties of the BFS-C in such aspects as construct validity, internal, and test–retest reliability among couples coping with CRC in China. Healthcare professionals need to treat couples as a unit and develop dyadic interventions to improve dyadic BF when supporting CRC survivors.

## 1. Introduction

Evidence shows that colorectal cancer (CRC) is one of the most common cancer types in the world in terms of incidence and mortality [1]. That is the case in China too, where both CRC incidence and mortality rates have demonstrated a progressively growing tendency since 1990 [2]. It has long been recognized that in the context of an exceedingly stressful experience, e.g., the diagnosis of cancer and its management, the unavoidable negative life fluctuations that follow (e.g., depressive and traumatic indications, impaired physical activities, reduced quality of life) [3,4,5] may also be accompanied by positive features [6].

Indeed, research findings have discovered that either cancer survivors or their intimate others experience positive life changes, e.g., intensified self-awareness, adjusted life precedence, and improved family interactions [7,8,9,10,11,12]. Under the circumstance of cancer-related stressful incidents, the positive experiences have been designated using numerous terminologies, including benefit finding (BF), positive effects, and post-traumatic growth [8]. The terminology BF was selected in the current study to describe the positive life experience that follows a CRC identification in CRC survivor–spousal caregiver (SC) couples [13].

Given the growing attention on and increasing acknowledgement of the prominence of BF in the context of cancer investigation, various types of scales assessing BF have been used [14]. However, it has been reported that in a majority of BF instruments, there is a need for further validation of their psychometric properties [14]. Taking the 17-item Benefit Finding Scale (BFS) [15] as an example, despite having served as one of the most frequently used BF measures in a wide variety of cancers, the evidence for its construct validity remains inconsistent [11,13,15,16,17,18,19,20], with either unidimensional [15,16,17] or multidimensional [11,13,18,19,20] models reported. These discrepancies in the 17-item BFS indicate the need to further validate its psychometric properties. Considering the viewpoint that an illness-specific tendency on BF finding exists, that is, findings of BF may not be transferrable across various illnesses, e.g., among different cancer types in the present situation [21], it is essential to examine its psychometric properties under the circumstance of a particular cancer category, e.g., CRC.

Further, evidence corroborates the view that the challenges following a cancer identification and the succeeding related cancer management plan involves the cancer family, e.g., patient–family caregiver dyads, particularly cancer couples, in coping together and supporting one another throughout the entire cancer trajectory [22]. The fact that couples cope with cancer together is increasingly recognized [23,24,25], with diverse types of dyadic relationships, including role alteration, dyadic interaction, and relationship or marriage quality [26,27,28]. Therefore, it is reasonable to conclude that a need exists to explore the related aspects from a dyadic perspective in a cancer context.

To fill in the above illustrated study gaps, the current study was designed to measure and estimate BF from the dyadic perspective of couples coping with CRC together. Based on our previous study [13], which was mainly dedicated to discovering the factor analysis of the Chinese version 17-item BFS (BFS-C) using samples of 772 dyads of mixed cancer (any type of cancer) patients and their family caregivers, the present study is psychometrically a more in-depth data analysis and exploration of the benefits that were experienced using specific examples of CRC survivor–SC dyads. The specific aims were twofold: (i) to examine the psychometric properties of the BFS-C in terms of construct validity, internal, and test–retest reliability, and (ii) to explore the experienced benefits in terms of BF levels and their correlations with sociodemographic variables in CRC survivor–SC dyads.

## 2. Methods

### 2.1. Participants and Procedures

Present data are drawn from a study (with details of the participants and procedures) exploring “the dyadic relationship of BF and its impact on quality of life in colorectal cancer survivor and spousal caregiver couples” [29]. Briefly, participants included CRC survivors who suffered from CRC and had completed first-line active treatment, and their partner or spouse: who took care of their spouse with CRC. The targeted sample was confirmed by means of the prerequisite of conducting factor analysis, e.g., confirmatory factor analysis (CFA) in this case, with a recommendation of 200–400 cases in most models [30]. The present analysis only involves the psychometric properties of the BFS-C and the experienced benefits in terms of BF levels and their correlations with the sociodemographic variables in CRC survivor–SC dyads.

After receiving ethical approval from the associated research ethics board (no. HREC201804001), the head nurse of the oncology ward approached eligible couples and invited them to take part in the survey. Once written informed consent was received from the targeted couples, they were advised to finish the survey independently. The investigation was conducted between May 2018 and December 2018 at a hospital in China. To evaluate test–retest reliability, 40 couples were designated for a second assessment (with approximately two to three weeks between the two assessments).

### 2.2. Assessment Measurements

Assessment measurements included a self-developed demographic questionnaire and the 17-item BFS [15], with the former applied to solicit demographic and health-correlated information, and the latter the BF. The BFS, including 17 items, has been evaluated in populations of cancer patients [11,13,15,16,17,18,19] and family caregivers [11]. For the BFS-C, a previous study has established a three-factor construct validity in dyads of Chinese cancer patients and family caregivers [13].

### 2.3. Data Analysis

A CFA was conducted on the BFS-C to further approve its previous three-factor model using Amos version 22.0 (IBM, Armonk, New York, USA). Table 1 shows data analysis for psychometric properties, including method applied and related indices and values for adequate model in CFA for construct validity [31], values of Cronbach’s α for estimating internal reliability, correlation coefficient (r), and Kappa for test–retest reliability at the scale and item levels, respectively [32].

Paired *t*-tests were conducted for comparison of CRC survivor scores to SC scores using r as effect size measures for correlations. Cutoff standards relating to the prevalence of the BFS-C at an item level (scoring 4 or 5) were prearranged along with a previous description by Llewellyn et al. [19]. In addition, according to the responses of the scale labels, where “not at all” = 1, “a little” = 2, “moderately” = 3, “quite a bit” = 4, and “extremely” = 5, scoring “1 or 2”,“3”, and “4 or 5” were considered low, moderate, and high levels of BF, respectively. In this way, high levels of BF equal the above cutoff standards of relating to the prevalence of the BFS-C (scoring 4 or 5) [19].

Pearson correlations, *t*-tests, and one-way ANOVAs were performed to examine the correlations of overall BFS scale level with the sociodemographic variables, e.g., age, gender, education, and working status, in CRC survivors and SCs. The Statistical Package for the Social Sciences, version 22.0 (SPSS, Chicago, Illinois, USA) was employed to conduct data analysis with the exception of CFA, which was conducted using Amos version 22.0.

## 3. Results

As shown in Table 2, a total of 286 couples had an average age of approximately 60 (ranging from 28–83) years old, with nearly 24 months on average since diagnosis for CRC survivors. The majority of CRC survivors were male (62.6%). Only about 10% of couples (11.9 and 8.7% for CRC survivors and SCs, respectively) had an educational level of a university undergraduate degree or above. Most participants were not working. Thirty-four of the 40 couples completed a second evaluation, which assessed test–retest reliability.

### 3.1. Psychometric Properties of the BFS-C

**Figure 1:**
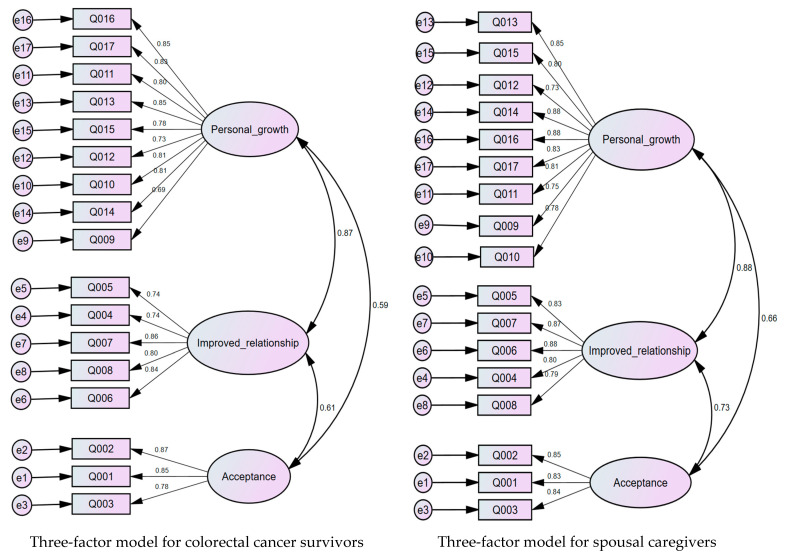
BFS-C structure model with standardized path coefficients for colorectal cancer survivors and spousal caregivers.

Construct validity: CFA of the BFS-C presented that the three-factor structure fit the data reasonably well, while values of CMIN/DF, RMSEA, SRMR, and CFI equaled 2.742, 0.079, 0.0467, and 0.949, respectively, for CRC survivors, while values of CMIN/DF, RMSEA, SRMR, and CFI for SCs were 2.793, 0.080, 0.0462, and 0.953, respectively. Figure 1 illustrates CFA standardized path coefficients.

Internal reliability: As shown in Table 3, good to excellent item–factor correlations were identified for both CRC survivors (r = 0.665–0.830, column 2) and SCs (r = 0.724–0.850, column 5). The Cronbach’s α of overall scale and each subscale ranged from 0.870 to 0.951 for CRC survivors (column 3), and from 0.876 to 0.959 for SCs (column 6).

Test–retest reliability: At the scale level, the test–retest correlations ranged from good to excellent for CRC survivors (r = 0.752–0.922, Table 3: column 4), and from moderate to good for their SCs (r = 0.469–0.654, Table 3: column 7). At the item level, the average Kappa coefficient was 52 and 46% for CRC survivors and SCs, respectively, with most items (15 out of 17 for CRC survivors, and 11 out of 17 for SCs) having a Kappa coefficient greater than 40% (Table 3: columns 4 and 7).

### 3.2. Experienced Benefits under Cancer Care between CRC Survivors and SCs

As presented in Table 4, at the item level, the percentage of participants reporting a low level of BF ranged from 15.4 to 36.4% in CRC survivors (column 2), and from 13.3 to 35.9% in SCs (column 6). The percentage of participants reporting a moderate BF level ranged from 19.2 to 27.3% in CRC survivors (column 3), and from 17.3 to 24.9% in SCs (column 7). While the high BF level is the same as the endorsement of positive growth in the item level (scoring 4 or 5), the percentage of participants reporting a BFS-C positive growth experience ranged from 43.0 to 63.6% in CRC survivors (column 4), and from 43.5 to 65.3% in SCs (column 8). Overall, SCs experienced higher levels of the BFS-C than CRC survivors, with two exceptions, in items 14 and 15. However, only two statistically significant differences were identified, in items 5 (*p* < 0.05) and 10 (*p* < 0.01). Effect size (r) measures for correlations ranged from 0.332 to 0.575 at the item level, and r = 0.612 at the overall scale level.

Further analysis of the correlations of overall BFS level with the sociodemographic variables in CRC survivors and SCs showed that (Table 5) ① BF of CRC survivors was negatively related to CRC survivors age (r = −0.14, *p* = 0.021, Table 5a), with CRC survivors who were older more likely to report lower levels of BF; ② significant differences in BF (*p* = 0.001) of CRC survivors in terms of education level, with a trend that CRC survivors with higher education reported higher levels of BF (Table 5a); ③ significant differences in SC BF in terms of the education levels of both CRC survivors (*p* = 0.035, Table 5a) and SCs (*p* = 0.005, Table 5b), with a trend that the higher the education level, the higher the BF scores; ④ significant differences in SC BF in terms of SC time spent in caring for the CRC survivors per day (*p* = 0.010, Table 5b), with a trend that the longer the time spent by the SC in caring for the CRC survivors per day, the higher the BF scores.

## 4. Discussion

The overall objectives of this paper involved examining the psychometric property of the BFS-C and exploring the experienced benefits in CRC survivor–SC dyads. Our findings indicate that the three-factor construct fit the data reasonably well, the BFS-C has good internal consistency, with an overall moderate level of agreement for test–retest reliability. There is also evidence that effect size ^®^ measures exist for correlations ranging from 0.332 to 0.575 at the item level, and r = 0.612 at the overall scale level. Grounded in the study aims and findings, the discussion mainly focuses on the following two aspects: the psychometric properties of the BFS-C and experienced benefits under cancer care between CRC survivors and SCs.

### 4.1. The Psychometric Properties of the BFS-C

The current CFA analysis further confirms the three-factor structure of the BFS-C [13]. As we had mentioned previously, evidence for the construct validity of the 17-item BFS remains inconsistent, with both unidimensional [15,16,17] and multidimensional [11,13,18,19,20] structures identified in related research. These discrepancies may partly be due to patients coming from different cultural backgrounds, as well as having different contexts for their cancer diagnosis, e.g., a one-factor structure for breast cancer patients in the United States [15], as well as for prostate cancer patients in Australia [16]; a four-factor model for mixed cancer patients in Germany [18]; a five-factor model for breast cancer populations in China [20], and a six-factor model for caregivers of American mixed cancer patients [11]. Accordingly, it is suggested that reporting at the item and/or overall scale levels be applied for the appraisal of patients across various cultural backgrounds and with diverse cancer diagnoses, whereas reporting on the different factor structure levels could be used in an in-depth national analysis.

Evidence from the present sample showed that the BFS-C had good internal consistency (with all Cronbach’s α ≥ 0.870). This good internal consistency of the BFS-C is in line with other reports, either in samples of patients with cancer, as well as in family caregivers (Cronbach’s α = 0.76–0.96) [11,13,15,16,17,18,19,20].

In terms of test–retest reliability, current findings showed a high and moderate level of agreement at the scale level (including the total and subscale scores) and at the item level, respectively, as suggested by Portney and Watkins [32]. The high level of agreement in the total scale level is in line with a report on samples of breast cancer patients [15]. No similar report on test–retest reliability for SCs was identified. Further investigation is required to establish its test–retest reliability in cancer practice.

### 4.2. Experienced Benefits under Cancer Care between CRC Survivors and SCs

Our findings on CRC survivors experiencing a percentage of positive growth (BF in this case) on 17 item levels (ranging from 43.0 to 63.6%) are somewhat in line with another study, in that cancer patients reported a percentage range of positive growth, from 33 to 85%. SCs in the present sample reported similar rates of positive growth (ranging from 43.5 to 65.3%) as CRC survivors. Although no similar report was identified for SCs of cancer survivors, the above findings of couples experiencing positive growth are in line with our previous findings on cancer patient–caregiver dyads [33].

Interestingly, both CRC survivors and their SCs reported their lowest and highest BF experience in the same items, i.e., item 12 and item 4, respectively, see Table 4). This may be a reminder that coping with the disease brought families closer together and improved their relationships. On the other hand, more attention also needs to be given to social support, e.g., support from friends, neighbors, and the surrounding community [10,11,12,27,28,29].

Further comparisons on the mean value of paired differences between CRC survivor and SC couples found that SCs experienced greater levels of BF in items 5 and 10 than CRC survivors did. This may be due to the demands of the caregiver role in caring for loved ones with cancer, e.g., physical (symptom management), mental (emotional), social (their family), as well as financial [34].

Findings that older CRC survivors were more likely to report lower levels of BF is a reminder that more attention should be given to elderly CRC survivors populations. Findings that CRC survivors with higher education reported higher levels of BF is consistent with the findings of another study, in that greater educational achievement was found to have a protective effect on BF [19]. It is assumed that CRC survivors who had received higher education can easily comprehend the disease and its treatment, which could facilitate their coping process and increase the benefits they experience. However, a different finding was reported by Jansen et al. [7], in that CRC survivors with higher education levels were associated with less BF. These inconsistent findings on the association between education levels and BF require future verification.

In addition, no similar report was identified regarding the associations of BF in SCs with other variables, e.g., education levels of both CRC survivors and SCs, time spent by SCs in caring for CRC survivors per day. This is a reminder that more attention should be paid to SCs with lower education levels, who spent a shorter time caring for a CRC survivor per day. It is suggested that special support be provided to couples with lower education levels.

In the Chinese culture, due to the Confucian ideal of filial piety, caregiving is considered an essential and integral element of family life [35]. SCs of cancer patients would sacrifice their own health to take care of their loved one with cancer [36]. In addition, the dual aspect of mutual “protection” in couples coping with cancer [36] further increases the caregiving burden. A previous qualitative study on Chinese couples coping with CRC also showed that while providing support to one another, couples must manage various challenges, e.g., insufficient communication, lack of knowledge, role conflict, and financial burden. The couples stated that receiving help or support from healthcare professionals would facilitate their journey of coping with CRC together and providing mutual support to one another [28]. Thus, it is of paramount importance for healthcare professionals to provide support to couples in their journey of coping with cancer together, and to encourage them to experience the benefits of the journey, while also confronting the challenges.

### 4.3. Limitations

As this study includes only Chinese couples dealing with CRC, which can be viewed as a very selective sample, its findings should be generalized only with the utmost caution to CRC patients from other cultural backgrounds and cancer diagnoses other than CRC. Further exploration, targeting populations coping with diverse cancer types in different cultures, should be conducted. Moreover, although the extracted sample size was confirmed by the prerequisite to conduct factor analysis, the second survey’s small sample size for assessing test–retest reliability may act as another study limitation. Further evaluation using an adequate sample size is required. In addition, the majority prevalence of males versus females in this study could have influenced the results. Future study on a balanced gender population is needed. Another limitation could be that it is necessary to study the concurrent validity with other tests assessing BF or personal growth.

## 5. Conclusions

The current findings not only confirm the three-factor construct validity of the BFS-C, but also offer the acceptable psychometric properties of the BFS-C in Chinese CRC survivor and SC couples. Considering the BF psychological properties, it is suggested that while adopting this instrument in clinical practice, it would be beneficial to apply other possible instruments and understand the possible correlations with other psychological wellbeing surveys. In addition, the findings also demonstrate that a dyadic BF relationship in couples coping with CRC may exist. Further intervention studies on improving dyadic BF in couples coping with CRC are highly recommended, particularly for those with low or moderate BF levels and lower education levels.

## Figures and Tables

**Table 1:** Data analysis for psychometric properties.

Psychometric Properties	Method	Indices and Values
Construct validity	Confirmatory factor analysis	CMIN/DF ≤ 3 RMSEA ≤ 0.08 SRMR ≤ 0.08 CFI ≥ 0.90
Internal reliability	Cronbach’s α	Cronbach’s α ≥ 0.75
Test–retest reliability	Scales level: Pearson correlations	Values of the correlation coefficient (r): <0.25: little relationship 0.25–0.50: fair relationship 0.50–0.75: moderate to good relationship >0.75: excellent relationship
	Item level: Kappa coefficient	Values of Kappa: <40%: poor to fair agreement 41 to 60%: moderate agreement 61 to 80%: excellent agreement >80%: substantial agreement

Note: CMIN/DF = ratio of Chi-square to df; RMSEA = a root mean square error of approximation; SRMR = the standardized root mean squared residual; CFI = a confirmatory fit index.

**Table 2:** Descriptive statistics of colorectal cancer survivors and spousal caregivers (*n* = 286).

Characteristics	CCS, *n* (%) *	SC, *n* (%) *
Age (mean ± SD), years	59.8 ± 10.5 (ranging from 28–83)	59.2 ± 10.4 (ranging from 28–83)
Gender		
Male	179 (62.6)	107 (37.4)
Female	107 (37.4)	179 (62.6)
Level of education		
Primary school or less	175 (61.0)	158 (55.2)
High school	76 (26.6)	103 (36.0)
University or above	34 (11.9)	25 (8.7)
Working status		
Working	62 (21.7)	75 (26.2)
Not working	222 (77.6)	208 (72.7)
Average time since diagnosis/duration in their role as a SC	23.9 ± 19.0 months (ranging from 10–132 months)	<6 months: 5 (1.7) 6 months ~2 years: 212 (74.1) >2 years ~5 years: 46 (16.1) >5 years: 22 (7.7)
Couples were informed about the disease **		
Partly informed	84 (29.3)	57 (19.9)
Well informed	201 (70.3)	226 (79.0)
Time spent by SC in caring for patients/day [in hours, *n* (%)]		<2 h: 22 (7.7) 2~4 h: 43 (15.0) >4~6 h: 35 (12.2) >6~8 h: 41 (14.3) >8 h: 143 (50.0)
Benefit finding in BFS (mean ± SD)		
Overall scale	60.1 ± 15.2 (range: 17–85)	61.5 ± 15.7 (range: 17–85)
F1: Personal growth	31.2 ± 8.9 (range: 9–45)	31.7 ± 9.1 (range: 9–45)
F2: Improved relationship	18.5 ± 4.9 (range: 5–25)	19.0 ± 4.9 (range: 5–25)
F3: Acceptance	10.4 ± 3.0 (range: 3–15)	10.7 ± 3.1 (range: 3–15)

Note: CCS = colorectal cancer survivors; BFS = the 17-item Benefit Finding Scale; SC = spousal caregivers; SD = standard deviation. * The total *n* does not equal 286 because of missing value. ** Well informed: the CCS fully understood his/her condition; or the SC was well informed about his/her spouse’s disease; Partly informed: the CCS was informed about the diagnosis of cancer, but not about the severity of his/her condition; or the SC was partly informed about his/her spouse’s disease.

**Table 3:** Cronbach’s alphas for each factor and item–factor correlations, test–retest reliability (*n* = 286).

Factors/item Description	Results of Colorectal Cancer Survivors			Results of Spousal Caregivers		
Having Had Cancer Has (for Colorectal Cancer Survivors) Having Provided Care for the Survivor through His/Her Cancer Experience Has (for Spousal Caregivers)	Item–Factor Correlation	Cronbach’s Alpha if Item Deleted	Test–Retest Reliability (*n* = 34) ^†^	Item–Factor Correlation	Cronbach’s Alpha if Item Deleted	Test–Retest Reliability (*n* = 34) ^†^
**Factor 1: Personal growth**		**0.938**	0.922 **		**0.946**	0.654 *
16. Helped me become more focused on priorities, with a deeper sense of purpose in life	0.822 **	0.926	0.413	0.846 **	0.934	0.388
17. Helped me become a stronger person, more able to cope effectively with future life challenges	0.785 **	0.928	0.485	0.791 **	0.938	0.359
11. Led me to deal better with stress and problems	0.756 **	0.930	0.581	0.776 **	0.938	0.526
13. Contributed to my overall emotional and spiritual growth	0.830 **	0.925	0.575	0.832 **	0.935	0.386
15. Helped me realize who my real friends are	0.759 **	0.929	0.711	0.787 **	0.938	0.437
12. Led me to meet people who have become some of my best friends	0.723 *	0.931	0.478	0.724 *	0.941	0.369
10. Taught me to be patient	0.765 **	0.929	0.547	0.740 *	0.940	0.538
14. Helped me become more aware of the love and support available from other people	0.769 **	0.929	0.610	0.850 **	0.934	0.473
9. Made me more aware and concerned for the future of all human beings	0.665 *	0.936	0.582	0.733 *	0.942	0.427
**Factor 2: Improved relationship**		**0.892**	0.752 **		**0.917**	**0.469**
5. Made me more sensitive to family issues	0.713 *	0.874	0.514	0.788 **	0.897	0.361
4. Brought my family closer together	0.694 *	0.878	0.683	0.748 *	0.905	0.574
7. Shown me that all people need to be loved	0.793 **	0.855	0.570	0.830 **	0.888	0.427
8. Made me realize the importance of planning for my family’s future	0.722 *	0.872	0.458	0.733 *	0.909	0.378
6. Taught me that everyone has a purpose in life	0.759 **	0.863	0.339	0.834 **	0.888	0.541
**Factor 3: Acceptance**		**0.870**	0.802 **		**0.876**	0.651 *
2. Taught me how to adjust to things I cannot change	0.770 **	0.798	0.260	0.781 **	0.806	0.538
1. Led me to be more accepting of things	0.775 **	0.794	0.414	0.755 **	0.831	0.601
3. Helped me take things as they come	0.707 *	0.855	0.588	0.748 *	0.837	0.561
**Overall scale**		**0.951**	0.882 **		**0.959**	0.592 *

* Moderate to good correlation (0.50 < r < 0.75). ** High correlation (r ≥ 0.75). ^†^ The test–retest correlations at the scale level, the Kappa coefficient at the item level. Bold: highlight the result of each factor.

**Table 4:** Percentage of participants reporting growth experience in 17-item level, mean values, paired samples differences, and Pearson correlations between colorectal cancer survivors and spousal caregivers in 17-item Benefit Finding Scale (*n* = 286).

Benefit Finding Scale Item	Colorectal Cancer Survivors				Spousal Caregivers				t	r
Having Had Cancer Has… (for Colorectal Cancer Survivors) Having Provided Care for the Survivor through His/Her Cancer Experience Has (for Spousal Caregivers)	% ^†^	% ^†^	% ^†^	Mean (SD)	% ^†^	% ^†^	% ^†^	Mean (SD)		
1. Led me to be more accepting of things	25.2	27.3	47.6	3.40(1.11)	25.9	19.2	54.9	3.52(1.20)	−1.772	0.463 ***
2. Taught me how to adjust to things I cannot change	26.6	26.2	47.2	3.37(1.13)	26.2	21.3	52.4	3.47(1.18)	−1.278	0.332 ***
3. Helped me take things as they come	17.5	26.7	55.8	3.60(1.12)	18.6	22.1	59.3	3.68(1.13)	−1.135	0.432 ***
4. Brought my family closer together	15.4	21.0	63.6	3.77(1.11)	13.3	21.4	65.3	3.89(1.10)	−1.871	0.497 ***
5. Made me more sensitive to family issues	20.1	22.6	57.2	3.63(1.21)	15.1	21.8	62.7	3.80(1.13)	−2.227 *	0.372 ***
6. Taught me that everyone has a purpose in life	20.3	21.7	58.0	3.62(1.18)	16.1	24.6	59.3	3.75(1.12)	−1.914	0.450 ***
7. Shown me that all people need to be loved	17.0	19.8	63.0	3.76(1.18)	16.5	19.6	63.9	3.83(1.14)	−1.126	0.495 ***
8. Made me realize the importance of planning for my family’s future	19.9	19.2	60.8	3.70(1.25)	18.6	20.0	61.4	3.72(1.22)	−0.319	0.445 ***
9. Made me more aware and concerned for the future of all human beings	36.4	19.2	44.4	3.12(1.40)	35.9	17.3	46.8	3.20(1.42)	−1.096	0.575 ***
10. Taught me to be patient	22.8	20.7	56.5	3.55(1.16)	17.1	21.3	61.5	3.74(1.14)	−2.627 **	0.481 ***
11. Led me to deal better with stress and problems	16.5	26.1	57.4	3.65(1.08)	16.2	24.9	58.9	3.72(1.12)	−1.036	0.399 ***
12. Led me to meet people who have become some of my best friends	23.6	23.4	43.0	3.18(1.22)	33.0	23.5	43.5	3.27(1.24)	−1.311	0.508 ***
13. Contributed to my overall emotional and spiritual growth	28.3	24.1	47.6	3.33(1.20)	28.4	24.9	46.7	3.40(1.21)	−0.945	0.457 ***
14. Helped me become more aware of the love and support available from other people	18.9	24.1	57.0	3.68(1.14)	21.1	23.6	55.3	3.60(1.16)	1.189	0.503 ***
15. Helped me realize who my real friends are	24.1	23.1	52.8	3.52(1.21)	28.8	21.4	49.8	3.45(1.27)	0.878	0.519 ***
16. Helped me become more focused on priorities, with a deeper sense of purpose in life	25.5	21.7	52.8	3.46(1.25)	24.2	21.1	54.7	3.54(1.23)	−1.016	0.515 ***
17. Helped me become a stronger person, more able to cope effectively with future life challenges	20.7	21.4	57.9	3.65(1.24)	17.2	22.1	60.7	3.77(1.14)	−1.519	0.450 ***
Overall scale				3.54(0.89)				3.61(0.92)	−1.461	0.612 ***

Note: SD = standard deviation; r = effect size measures for correlations. ^†^ Scoring 1 or 2/3/4 or 5 was considered to be a low/medium/high level of benefit finding, respectively. Scoring 4 or 5 was considered an endorsement of positive growth in item level. * *p* < 0.05; ** *p* < 0.01; *** *p* < 0.001.

**Table 5:** Correlations of overall Benefit Finding Scale level with sociodemographic variables in colorectal cancer survivors and spousal caregivers (*n* = 286).

Variables	BF of CRC Survivors				BF of Spousal Caregivers			
	*n* *	Mean(SD)	t/F/r **	*p*-Value	*n* *	Mean(SD)	t/F/r **	*p*-Value
**a: CRC survivor-related variables**								
Age	280		−0.140	0.021	280		−0.007	0.906
Gender								
Male	174	60.47(14.74)			176	62.02(15.35)		
Female	103	59.46(15.87)	0.533	0.594	106	60.72(16.20)	0.670	0.504
Levels of education								
Primary school or less	168	58.28(15.38)			174	60.94(15.86)		
High school	74	60.28(14.24)			74	59.88(15.09)		
University or above	34	68.59(13.54)	6.806	0.001	33	68.00(14.80)	3.402	0.035
Working status								
Working	60	62.13(13.82)			61	61.31(15.14)		
Not working	215	59.69(15.28)	1.118	0.265	219	61.67(15.75)	−0.157	0.239
Time since diagnosis	263		0.004	0.946	263		−0.021	0.738
Informed about the disease								
Partly informed	80	57.60(15.34)				61.62(15.63)		
Well informed	196	61.17(15.01)	−1.783	0.076		61.56(15.73)	0.031	0.975
**b: SC-related variables**								
Age	275		−0.045	0.456			−0.068	0.264
Gender								
Male	103	59.47(15.87)			106	60.72(16.20)		
Female	174	60.47(14.74)	−0.533	0.594	176	62.02(15.35)	−0.670	0.504
Levels of education								
Primary school or less	152	58.82(15.65)			156	59.32(16.37)		
High school	100	60.48(14.06)			102	62.51(14.32)		
University or above	25	66.32(15.15)	2.712	0.068	24	70.38(13.29)	5.466	0.005
Working status								
Working	76	62.66(15.22)			73	62.33(14.84)		
Not working	201	59.06(15.09)	1.739	0.083	206	61.28(15.88)	0.492	0.623
Duration in their role as a spousal caregiver								
<6 months	4	63.75(10.01)			4	62.50(12.07)		
6 months~2 years	205	60.09(15.27)			210	61.44(15.88)		
>2 years~5 years	46	63.78(15.24)			46	63.98(15.81)		
>5 years	21	53.42(9.12)	2.427	0.066	21	58.29(12.51)	0.682	0.564
Informed about the disease								
Partly informed	55	61.71(14.25)			56	63.14(15.37)		
Well informed	219	59.85(15.40)	0.810	0.419	223	61.37(15.69)	0.760	0.448
Time spent by SC in caring for patient/day								
<2 h	22	54.77(15.65)			22	53.77(16.82)		
2~4 h	42	57.14(17.11)			43	56.44(17.00)		
>4 h~6 h	33	58.42(13.45)			33	61.81(12.48)		
>6 h~8 h	38	61.18(11.58)			41	63.14(14.16)		
>8 h	140	61.85(15.44)	1.722	0.145	141	63.76(15.66)	3.418	0.010

Note: BF = benefit finding; CRC = colorectal cancer; SC = spousal caregivers; SD = standard deviation. * The total *n* does not equal 286 because of missing value. ** r/t/F: r = one group using person correlation; t = two groups using t-test; F = above two groups using one-way ANOVA.

## Data Availability

We (the authors) have full control of all primary data and agree to allow the journal to review the data if requested.
